# Atherosclerosis evaluation and cardiovascular risk estimation using coronary computed tomography angiography

**DOI:** 10.1093/eurheartj/ehae190

**Published:** 2024-04-12

**Authors:** Nick S Nurmohamed, Alexander R van Rosendael, Ibrahim Danad, Quyen Ngo-Metzger, Pam R Taub, Kausik K Ray, Gemma Figtree, Marc P Bonaca, Judith Hsia, Fatima Rodriguez, Alexander T Sandhu, Koen Nieman, James P Earls, Udo Hoffmann, Jeroen J Bax, James K Min, David J Maron, Deepak L Bhatt

**Affiliations:** Department of Cardiology, Amsterdam UMC, Vrije Universiteit Amsterdam, Amsterdam, The Netherlands; Department of Vascular Medicine, Amsterdam UMC, University of Amsterdam, Amsterdam, The Netherlands; Division of Cardiology, The George Washington University School of Medicine, Washington, DC, United States; Department of Cardiology, Leiden University Medical Center, Leiden, The Netherlands; Department of Cardiology, University Medical Center Utrecht, Utrecht, The Netherlands; Department of Cardiology, Radboud University Medical Center, Nijmegen, The Netherlands; Department of Health Systems Science, Kaiser Permanente Bernard J. Tyson School of Medicine, Pasadena, CA, United States; Section of Cardiology, Department of Medicine, University of California, San Diego, CA, United States; Department of Primary Care and Public Health, Imperial College London, London, United Kingdom; Faculty of Medicine and Health, University of Sydney, Australia, St Leonards, Australia; Department of Medicine, University of Colorado School of Medicine, Aurora, CO, United States; Department of Medicine, University of Colorado School of Medicine, Aurora, CO, United States; Department of Medicine, Stanford University School of Medicine, Stanford, CA, United States; Department of Medicine, Stanford University School of Medicine, Stanford, CA, United States; Department of Medicine, Stanford University School of Medicine, Stanford, CA, United States; Cleerly, Inc., Denver, CO, United States; Department of Radiology, The George Washington University School of Medicine, Washington, DC, United States; Cleerly, Inc., Denver, CO, United States; Department of Cardiology, Leiden University Medical Center, Leiden, The Netherlands; Cleerly, Inc., Denver, CO, United States; Department of Medicine, Stanford University School of Medicine, Stanford, CA, United States; Mount Sinai Fuster Heart Hospital, Icahn School of Medicine at Mount Sinai, 1 Gustave Levy Place, Box 1030, New York, NY 10029, United States

**Keywords:** Coronary artery disease, Atherosclerotic cardiovascular disease, Coronary computed tomography angiography, Major adverse cardiovascular events, Prevention

## Abstract

Clinical risk scores based on traditional risk factors of atherosclerosis correlate imprecisely to an individual’s complex pathophysiological predisposition to atherosclerosis and provide limited accuracy for predicting major adverse cardiovascular events (MACE). Over the past two decades, computed tomography scanners and techniques for coronary computed tomography angiography (CCTA) analysis have substantially improved, enabling more precise atherosclerotic plaque quantification and characterization. The accuracy of CCTA for quantifying stenosis and atherosclerosis has been validated in numerous multicentre studies and has shown consistent incremental prognostic value for MACE over the clinical risk spectrum in different populations. Serial CCTA studies have advanced our understanding of vascular biology and atherosclerotic disease progression. The direct disease visualization of CCTA has the potential to be used synergistically with indirect markers of risk to significantly improve prevention of MACE, pending large-scale randomized evaluation.

## Graphical Abstract

**Graphical Abstract:**
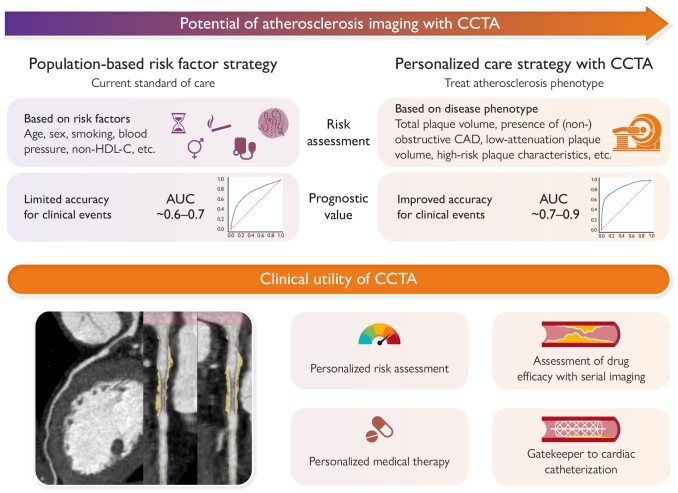
Coronary computed tomography angiography (CCTA) can change the population risk-based approach to a personalized care approach and provides important clinical utility. Coronary computed tomography angiography allows for precise atherosclerotic plaque quantification and characterization, and CCTA studies have advanced our understanding of vascular biology that holds potential to change our population risk-based approach to a personalized care approach. Coronary computed tomography angiography also provides clinical utility for assessment of drug efficacy and as a gatekeeper to cardiac catheterization lab. AUC, area under the curve; CAD, coronary artery disease; CCTA, coronary computed tomography angiography; HDL-C, high-density lipoprotein cholesterol.

## Background

The identification of patients at risk of cardiovascular events from atherosclerotic coronary artery disease (CAD) poses a major challenge in current prevention. Risk algorithms utilized in clinical practice, including the Systemic Coronary Risk Evaluation 2 (SCORE2) system and the Second Manifestations of ARTerial disease 2 (SMART2), Pooled Cohort Equations, and the Framingham Risk Score (FRS),^1–5^ are based on traditional cardiovascular risk factors and, while effective at a population level, precision for predicting events at an individual level is limited.^6–8^

### Limitations to current approaches in cardiovascular risk assessment: measurement of upstream and downstream indirect markers of heart disease rather than heart disease itself

Risk factors underpinning current clinical algorithms, including standard modifiable components of smoking, hypertension, diabetes, and hypercholesterolaemia, as well as age and sex, have been identified in large population studies. While these factors are associated with atherosclerosis progression/instability, they do not precisely integrate the dynamic complexity of pathophysiological processes that contribute to the development and progression of CAD. As a result, they cannot account for the heterogeneity in inter-individual atherogenic vulnerability or resilience. This is illustrated by poor discrimination and calibration in several validation studies.^8,9^ Indeed, many individuals develop atherosclerotic CAD, plaque rupture, and myocardial infarction without a single standard modifiable risk factor (SMuRF) meeting threshold for ‘action’.^10–14^

The fact that most asymptomatic patients remain unidentified before their myocardial infarction, and that more than two of three culprit plaques implicated in events are non-obstructive (<50% stenosis),^15,16^ highlights the need for reliable measures of atherosclerotic CAD itself. The inability to detect high-risk atherosclerosis is highlighted further by the many patients that experience out-of-hospital sudden cardiac death as their first and final cardiovascular event.^17^

The high event rates in symptomatic and secondary prevention patients can likely also be attributed to the absence of a reliable measure of atherosclerotic CAD itself—particularly of angiographically non-obstructive disease not detected by functional stress imaging. In symptomatic patients, non-invasive functional testing strategies, such as nuclear myocardial perfusion imaging using single-photon emission computed tomography (SPECT) or positron emission tomography (PET) are the gold standard for the non-invasive detection of myocardial ischaemia as an indirect marker for epicardial coronary stenosis and thus, need for revascularization. However, the majority of events occurs in patients with normal stress testing, highlighting the importance of detecting non-obstructive CAD for more effective prevention of major adverse cardiovascular events (MACE).^16^

The limitations of traditional risk estimation paradigms are an even more urgent problem considering the now globally rising deaths from cardiovascular diseases^18^ and the opportunity to stem this tide with novel preventive treatments to target different components of the atherosclerotic process, such as with monoclonal antibody PCSK9 inhibitors and inclisiran,^19^ icosapent ethyl,^20^ sodium-glucose co-transporter 2 (SGLT2) inhibitors,^21–23^ glucagon-like peptide-1 (GLP1) receptor agonists,^24–26^ bempedoic acid,^27^ low-dose rivaroxaban,^28^ and colchicine,^29^ amongst others. Due to the high costs associated with these medications and issues of polypharmacy as well as adherence, methods to distribute them to high-risk patients who are likely to derive the most benefit are needed.^30^

This review evaluates (i) the current use of coronary computed tomography angiography (CCTA) in clinical practice, (ii) its role in quantifying and characterizing CAD as the primary determinant for future MACE in symptomatic patients, (iii) its role in asymptomatic individuals or populations, and (iv) future directions for clinical trials investigating the role of CCTA in a personalized approach to prevent atherosclerotic cardiovascular disease (ASCVD).

## Non-invasive imaging for coronary artery disease: coronary computed tomography angiography

### Technological advancements enabling detailed coronary artery disease evaluation

Over the last 20 years, computed tomography (CT) scanners and techniques for CCTA have substantially improved, enabling more precise atherosclerotic plaque quantification and characterization. The first CT scanners used in large-scale CCTA trials were single-source 64-row detector scanners that had a isotropic spatial resolution of 0.625 mm and temporal resolution of 175 ms.^31^ The current third-generation, dual-source 2 × 192-row detector CT scanners can achieve a maximum spatial resolution of 0.24 mm and a temporal resolution up to 66 ms,^31^ while prospective triggering has drastically reduced the average radiation dose to below 3 mSv, equivalent to the amount of annual exposure due to background radiation for an individual living at sea level.^32^ Using a scanner with ≥320-row detector enables full-heart imaging within a single gantry rotation, and further reduces radiation dose (average ∼1 mSv) and eliminates step artefacts.^32,33^ In comparison to CCTA, median exposures are 13 mSv for SPECT,^34^ 4 mSv for PET,^34^ and 3 mSv for diagnostic cardiac catheterization.^35^ Most recently, novel photon-counting CT scanners have been introduced into clinical practice.^36^ These CT scanners measure the energy of individually counted X-ray photons resulting in a higher contrast-to-noise ratio, providing the ability to further reduce radiation exposure while improving spatial resolution and spectral imaging capabilities.

### Diagnostic accuracy of coronary computed tomography angiography for coronary atherosclerosis

The accuracy of CCTA for characterizing coronary atherosclerosis compared to intracoronary imaging has been validated in numerous studies. To detect any coronary plaque, compared to intravascular ultrasound (IVUS), a meta-analysis of 1360 patients from 42 studies showed a sensitivity and specificity of 93% and 92%, respectively, resulting in an area under the curve (AUC) of 0.97.^37^ A more recent study using 181 lesions from 151 patients reported an overestimation of lesion severity by CCTA compared to IVUS.^38^ Other studies reported differing degrees of over- and underestimation of lesion severity, most likely due to differences in lesion characteristics, lesion severity, and CCTA quality.^39–42^

Several studies also compared CCTA accuracy for luminal morphology using optical coherence tomography (OCT), showing good correlation despite the lower spatial resolution of CCTA.^43–45^ Findings with near-infrared spectroscopy (NIRS) have also been compared with plaque morphology observed with CCTA. The accuracy of CCTA-determined high-risk plaque for detecting NIRS lipid-rich plaque in 133 plaques from 47 patients was 94%, with a sensitivity of 93% and specificity of 94% (AUC 0.97).^46^

### Advantages and limitations of non-invasive coronary computed tomography angiography: comparison to invasive approaches

Coronary computed tomography angiography has several advantages over traditional invasive methods to visualize atherosclerosis (*Table 1*). First, CCTA enables ‘whole-heart’ coronary imaging, whereas invasive methods such as IVUS only allow imaging of a maximum of two thirds of the major epicardial vessels, and typically are performed on a single vessel. Second, CCTA allows for comprehensive characterization of CAD, offering the advantages of multiple invasive imaging modalities in a single non-invasive imaging examination, and additionally provides information on valvular and structural heart disease. As examples, IVUS is mainly used to estimate plaque burden, OCT is employed to determine vascular morphology and NIRS evaluates lipid-rich plaque. Finally, the major advantage of CCTA is that it is a safe procedure with negligible complication rates and low radiation burden, thereby allowing for serial measurement in a clinical setting, which is not feasible with traditional invasive procedures. Resulting from this non-invasive approach, CCTA costs are low (∼€280) and now on par with routine blood tests and considerably less expensive than nuclear stress testing. The introduction of fractional flow reserve from CCTA (FFR_CT_) has further extended the utility of CCTA to non-invasive assessment of coronary flow.^50–52^

**Table 1:** Advantages and disadvantages of coronary computed tomography angiography over traditional invasive approaches for atherosclerosis characterization

Measurement	CCTA	IVUS	OCT	NIRS
Requires invasive ICA	No	Yes	Yes	Yes
Whole-heart CAD assessment	+++	+	+	−
Plaque volume/burden	+++	+++	+	−
Positive remodelling	+++	+++	+	−
Plaque composition	++	++	+++	+
Calcium identification	+++	+++	+++	−
Calcium quantification	++	+	+++	−
Lipid core	++	+	++	+++
Thin-cap fibroatheroma	−	−	+++	−
Plaque rupture	−	+	+++	−
Intraluminal thrombus	+	++	+++	−
PCI guidance	+	+++	+++	−
Interobserver variability	++	++	+	+
Procedure costs (€)	∼280	∼1000 + ICA costs^47^	∼600 + ICA costs^48^	∼1000 + ICA costs^49^
Radiation dose^a^ (mSv)	1–3	−	−	−
Spatial resolution (mm)	0.6	0.15–020	0.012–0.015	0.1

^a^Radiation doses for IVUS, OCT, and NIRS are limited to the doses used during coronary angiography and are not increased by the invasive imaging modalities.

CAD, coronary artery disease; CCTA, coronary CT angiography; ICA, invasive coronary angiography; IVUS, intravascular ultrasound; OCT, optical coherence tomography; PCI, percutaneous coronary intervention; NIRS, near-infrared spectroscopy.

Importantly, CCTA is not without limitations compared to its invasive analogues: it possesses lower spatial and temporal resolution than invasive procedures. This precludes assessment of important atherosclerotic features, such as thin-cap fibroatheroma or macrophage infiltration of plaque. Given the recent introduction of photon-counting CCTA as well as deep learning reconstruction techniques that improve spatial resolution, the near-term future will ideally advance CCTA’s further contribution to the study of atherosclerosis.^53^ Additionally, CCTA is complicated in patients with a fast heart rate or arrhythmias and in patients with a low estimated glomerular filtration rate.

## The role of coronary computed tomography angiography in patients with symptoms or proven coronary artery disease

### Coronary computed tomography angiography-defined atherosclerosis for prediction of major adverse cardiovascular events in symptomatic patients

The burden of CCTA-defined extent of CAD is a strong predictor for MACE in patients with symptomatic or proven CAD, with consistent incremental prognostic value over the clinical risk spectrum as demonstrated in several subpopulations.^54–64^

A large body of studies has shown that the degree or extent of obstructive CAD (defined as either ≥50% or ≥70%) has important prognostic implications in patients undergoing clinically indicated CCTA.^65–68^ Compared with a normal CCTA, the multivariable adjusted hazard ratios (HR) for myocardial infarction or death were 2.2, 2.9, 3.5, and 4.7 for the presence of angiographically non-obstructive, one-vessel obstructive, two-vessel obstructive, and three-vessel or left main obstructive CAD in Coronary CT Angiography Evaluation For Clinical Outcomes: An International Multicenter (CONFIRM).^65^ In 17 793 patients from this registry, the two visually assessed CCTA parameters ‘number of proximal segments with mixed or calcified plaques’, and ‘the number of proximal segments with a stenosis ≥50%’ provided independent discriminatory value for death at 2.3-year beyond 3 commonly used clinical risk scores for future MACE, including the FRS.^69^

**Figure 1:**
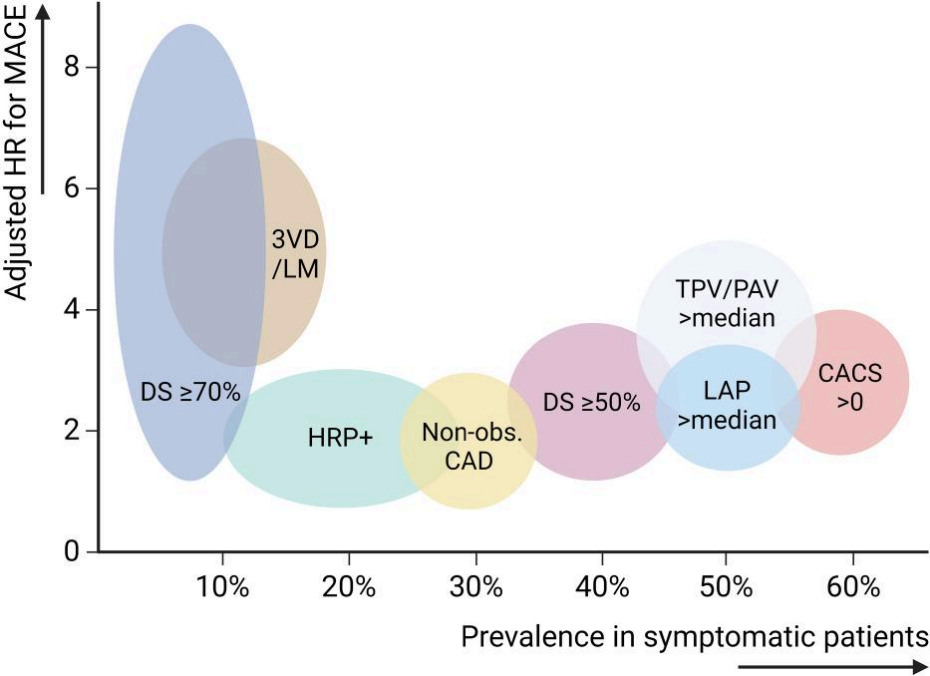
Prevalence and hazard of coronary computed tomography angiography characteristics in symptomatic patients. Estimates of the relative hazards and prevalence of different CCTA characteristics, derived from different large studies in symptomatic patients.^62–64,71–73^ 3VD, three-vessel disease; CACS, coronary artery calcium score; CCTA, coronary computed tomography angiography; DS, diameter stenosis; HR, hazard ratio; HRP+, presence of high-risk plaque; LAP, low-attenuation plaque; LM, left main disease; MACE, major adverse cardiovascular event; non-obs. CAD, non-obstructive coronary artery disease; PAV, percent atheroma volume; TPV, total plaque volume

Coronary atherosclerotic burden and stenosis severity are correlated, but atherosclerosis is the primary disease process while stenosis is a sequela of atherosclerosis in some patients; and, in the CONFIRM risk score, diffuseness of atherosclerosis dominated risk prediction over the severity of stenosis. The risk of MACE for non-obstructive CAD in >4 segments was equivalent to the risk of MACE for angiographically obstructive CAD.^70^ Similarly, increased risk for myocardial infarction or death was observed with every increasing diseased segment in patients without any obstructive stenosis.^71^ Nevertheless, as stenosis and atherosclerosis are strongly correlated in the majority of patients, studies incorporating the degree and extent of obstructive disease have shown similar predictive values for obstructive stenosis and the extent of non-obstructive CAD as indicators of total atherosclerosis burden (*Figure 1*).

**Figure 2:**
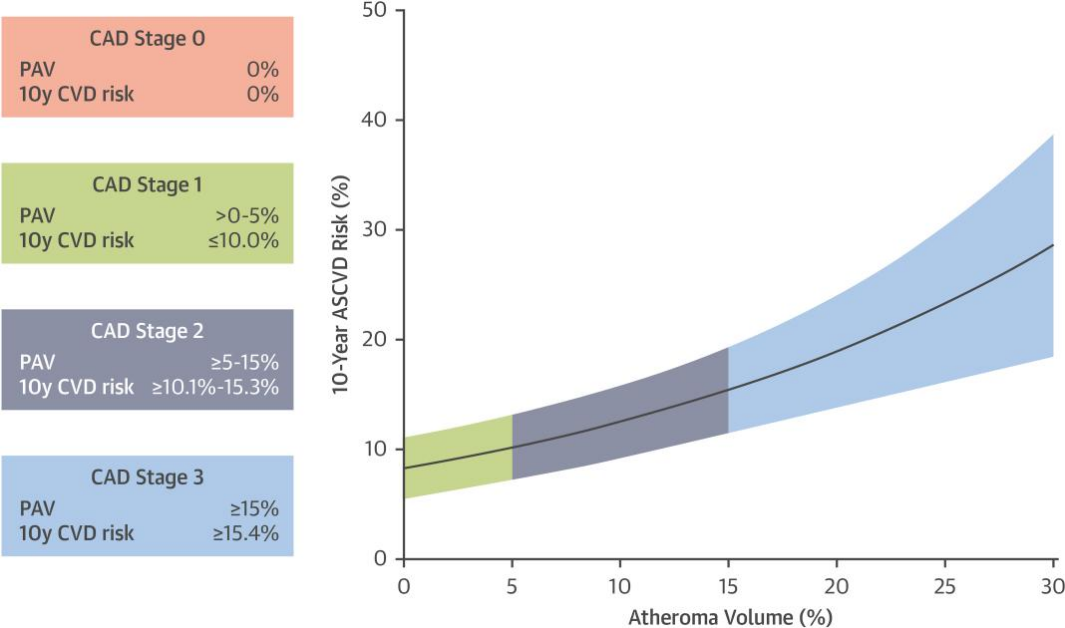
Relationship between coronary computed tomography angiography-derived plaque burden and 10-year risk for cardiovascular events. Ten-year risk of cardiovascular events according to different plaque stages based on percent atheroma volume. The risk for cardiovascular events increases with increasing plaque volume. ASCVD, atherosclerotic cardiovascular disease; CAD, coronary artery disease; CVD, cardiovascular disease; CCTA, coronary computed tomography angiography; PAV, percent atheroma volume. Adapted from Nurmohamed *et al.*^62^

Subsequent studies have investigated the role of coronary plaque characterization, including compositional assessment, vascular morphology, or positive remodelling of the artery, which has also been reported to yield prognostic utility in symptomatic patients or those suspected of CAD (*Figure 1*; *Table 2*). In the Incident Coronary Syndromes Identified by Computed Tomography (ICONIC), Scottish Computed Tomography of the Heart (SCOT-HEART), and Prospective Multicenter Imaging Study for Evaluation of Chest Pain (PROMISE) trials, plaque composition or plaque burden, next to the degree of coronary stenosis, was the strongest predictor of future acute coronary syndromes (ACS).^62–64,71,72^ These studies need to be interpreted in light of the degree of adjustment for overall CAD burden, which remains a stronger predictor than detailed morphological and compositional plaque characterization (*Figure 1*). In ICONIC, 234 patients who suffered core-lab verified ACS after baseline CCTA were matched to 234 patients not experiencing ACS after baseline CCTA, based on age, sex, risk factors, and stenosis severity (normal, non-obstructive, one-vessel, two-vessel, and three-vessel/left main obstructive CAD).^71^ In this cohort, total plaque volume was similar between cases and controls (289.7 mm^3^ ± 308.4 vs. 267.2 mm^3^ ± 285.7, *P* = .32), whilst the presence of severe stenosis (≥70%), the volume of low-density non-calcified plaque, and presence of high-risk plaque was higher in ACS cases. Low-density non-calcified, high-risk plaque, and severe stenosis had comparable HRs for ACS on a per-patient level (low-density non-calcified plaque: HR 1.44, high-risk plaque: HR 1.59, severe stenosis: HR 1.53). Additionally, diffuseness of disease, presence of high-risk plaque, and volume of fibro-fatty plaque was higher in culprit ACS cases. In analysis from the multicentre PROMISE trial in 4451 patients, the presence of obstructive CAD defined as a >70% stenosis or >50% left main stenosis was associated with an approximately nine-fold increased risk of MACE, defined as myocardial infarction, unstable angina, or death.^72^ Independent of luminal stenosis and ASCVD risk factors, but unadjusted for total plaque burden, which was not analysed in this study, high-risk plaque was associated with an additional modest increase in MACE risk [adjusted HR (aHR) 1.72, 95% confidence interval (CI) 1.13–2.62], predominantly in those without obstructive CAD.^72^ Furthermore, Williams *et al.*^64^ demonstrated the prognostic value of low-attenuation plaque independently of plaque burden, coronary artery calcium score (CACS) and presence of obstructive CAD in 1769 patients with stable chest pain undergoing CCTA in the SCOT-HEART trial. Low-attenuation plaque burden (<30 HU) was associated with an aHR of 1.60 per doubling (95% CI 1.10–2.34; *P* = .014) for myocardial infarction. In the same model, CACS was also associated with myocardial infarction [aHR 1.13 per doubling (95% CI 1.01–1.27); *P* = .041]. Also using external validation in SCOT-HEART, Lin *et al.*^63^ found that a total plaque volume above 238.5 mm^3^, as defined by deep learning plaque analysis, was associated with a more than five-fold increased risk of myocardial infarction [aHR 5.36 (95% CI 1.70–16.86); *P* = .0042]. Similarly, stratification based on total plaque volume also demonstrated a prognostic benefit beyond traditional CACS and clinical risk factors in a long-term 10-year outcomes study in 536 patients (*Figure 2*).^62^ Nevertheless, the highest plaque stage provided a similar HR to the presence of obstructive stenosis. Most recently, a *post hoc* analysis of the CORE320 study found no additional prognostic benefit of plaque burden quantification beyond CACS (AUC 0.64 for CAD staging vs. AUC 0.65 for CACS).^73^

**Table 2:** Studies evaluating independent prognostic value of detailed plaque characterization in symptomatic patients

Study	Design	Type of plaque quantification	Outcomes
Chang *et al.* (ICONIC)^71^	Case-control study, 234 patients with ACS propensity matched (based on clinical risk factors and CAD extent) to 234 patients without ACS	Quantitative evaluation of the total coronary tree. Low-density non-calcified plaque was defined by <30 HU.	Low-density non-calcified plaque (HR 1.44), high-risk plaque (HR 1.59), and severe stenosis (HR 1.53) were associated with a higher risk of ACS.
Ferencik *et al.* (PROMISE)^72^	RCT, 4451 symptomatic patients undergoing CCTA, followed for 25 months for death, MI, or unstable angina (*n* = 31)	Significant stenosis (>70% or >50% in left main) and high-risk plaque (positive remodelling, low computed tomographic attenuation, or napkin-ring sign) was visually assessed	Significant stenosis was associated with a 9-fold increase in MACE. Adjusted for significant stenosis and ASCVD risk score, patient-level presence of HRP was additionally associated with a modest risk increase [aHR 1.72 (95% CI 1.13–2.62)].
Williams *et al.* (SCOT-HEART)^64^	RCT, 1769 patients with stable chest pain undergoing CCTA, followed for 4.7 years for MI (*n* = 41)	Quantitative semi-automated evaluation of plaque burden and low-attenuation plaque	Adjusted for plaque burden, non-calcified and calcified plaque burden, CACS, the presence of visually obstructive CAD, and cardiovascular risk score, low-attenuation plaque was independently associated with MI [aHR 1.60 per doubling (95% CI 1.10–2.34); *P* = .014]. In the same model, CACS was also associated with MI [aHR 1.13 per doubling (95% CI 1.01–1.27); *P* = .041].
Lin *et al.* (SCOT-HEART)^63^	Multicentre observational study, external prognostic validation in SCOT-HEART, 1611 patients followed for 4.7 years for MI (*n* = 41)	Deep learning plaque analysis	Adjusted for stenosis and clinical risk factors, a total plaque volume of 238.5 mm^3^ or higher was associated with an increased risk of myocardial infarction (aHR, 5.36, [95%CI 1.70–16.86]; *P* = .0042).
Nurmohamed *et al.*^62^	Observational cohort study, 536 patients suspected of CAD, followed for 10 years for non-fatal MI, non-fatal stroke, revascularization and all-cause mortality (*n* = 116)	AI-guided plaque and stenosis quantification	Adjusted for clinical risk factors, stage 3 plaque based on PAV was associated with the composite outcome [aHR 3.57 (95% CI 2.12–6.00); *P* < .001], while obstructive stenosis >70% was associated with an aHR of 4.66 (95% CI 2.73–7.94).
Oeing *et al.* (CORE320)^73^	Observational cohort study, 372 symptomatic patients, followed for 4.9 years for cardiac death, MI, cardiac hospitalization, and late revascularization (*n* = 97)	Semi-automated plaque quantification	Atheroma burden, CAD-RADS, high-risk plaque provided no additional prognostic value beyond CACS (AUC 0.65 for CACS vs. 0.64 for CAD stage).

ACS, acute coronary syndrome; AI-QCT, Atherosclerosis Imaging-Quantitative Computed Tomography; CAD, coronary artery disease; CI, confidence interval; HU, Hounsfield units; HR, hazard ratio; MI, myocardial infarction; ICONIC, Incident Coronary Syndromes Identified by Computed Tomography; PAV, percent atheroma volume; PROMISE, Prospective Multicenter Imaging Study for Evaluation of Chest Pain; RCT, randomized clinical trial; SCOT-HEART, Scottish Computed Tomography of the Heart.

### Dynamic changes in coronary computed tomography angiography-defined atherosclerosis over time

Several studies have investigated the changes in CCTA-defined atherosclerosis over time, of which the PARADIGM (Progression of Atherosclerotic Plaque Determined by Computed Tomographic Angiography Imaging) has been the largest study to date.^74–76^ The PARADIGM registry included over 1000 patients with serial CCTA ≥2 years apart upon which atherosclerotic evaluation was performed. In PARADIGM, adjusted for baseline PAV, annualized increase in PAV was independently associated with MACE with a 23% increased risk per standard deviation increase during 8 years of follow-up, consistent in multiple subpopulations.^76,77^ For plaque progression, a clinically relevant threshold was defined by an increase of plaque volume associated with MACE, that is, annualized 1.0% increase in PAV.^76^ Specifically, patients experiencing MACE during follow-up previously had a three-fold higher progression rate compared with patients not experiencing MACE: 0.93% (IQR 0.34–1.96) vs. 0.32% (IQR 0.02–0.90; *P* < .001).^76^ Plaque progression evaluation using serial IVUS has yielded comparable results. Among combined data of six clinical trials utilizing serial IVUS, patients with MACE had an annual increase in PAV of 0.95% vs. 0.46% in patients without events (*P* < .001).^78^

### Serial coronary computed tomography angiography studies investigating the impact of medical therapy

Numerous studies have evaluated the impact of statin therapy on plaque burden and plaque composition.^79^ The PARADIGM registry involving 1255 patients who underwent serial CCTA, showed statin therapy to accelerate calcifications of non-calcified plaques, thus conferring plaque stabilization.^77^ Progression of calcified plaque was not associated with MACE, while in contrast rapid progression of non-calcified lesions was reflective of unstable CAD and associated with future adverse outcomes.^76^ The greatest reduction in non-calcified plaque volume was observed in a small but prospective randomized trial in HIV patients who were randomized to 20 mg atorvastatin vs. placebo. Non-calcified plaque volume decreased by 19% in the statin arm and increased by 20% during 1-year follow-up in the placebo arm. In addition, in comparison to the placebo group, patients with statin therapy had a lower total plaque burden, low-attenuation plaque volume and experienced a reduction in the number of plaques with positive remodelling after treatment.^80^ In line with this smaller study, a recent prespecified mechanistic substudy in 804 HIV-positive patients from the REPRIEVE trial found that pitavastatin significantly lowered non-calcified plaque volume compared with placebo (−1.7 mm^3^ vs. 2.6 mm^3^; *P* = .044).^81^ In parallel, the GLAGOV, PACMAN-AMI and HUYGENS studies have shown that, compared to placebo, PCSK9 inhibition reduced percent atheroma volume from IVUS by 1%, reduced lipid core burden and increased fibrous cap thickness.^82–84^ Similar results were found in a study using serial CCTA imaging.^85^ Both icosapent ethyl and colchicine have also shown to result in reductions in low-density or non-calcified plaque using serial CCTA (*Table 3*).^86–89^

**Table 3:** Studies investigating effect of medication on coronary computed tomography angiography-defined atherosclerosis progression

Intervention	Main inclusion criterion	Sample size	Duration of follow-up	Effect on plaque morphology
Statins^90^	Suspected or known coronary artery disease	857 patients with 2458 coronary lesions	3.6 years	Reductions in low-attenuation plaque (*β* = −0.02; *P* = .001) and fibro-fatty plaque (*β* = −0.03; *P* < .001) No change in fibrous plaque and low-density calcium Increase in high-density calcium and 1 K plaque
Atorvastatin^80^	HIV	19 patients receiving atorvastatin 21 patients receiving placebo	12 months	Reduction in non-calcified plaque volume compared to placebo (−19.4% vs. 20.4%; *P* = .009) Reduction in number of high-risk plaques compared to placebo
Pitavastatin^81^	HIV	402 patients receiving pitavastatin 402 patients receiving placebo	2 years	Reduction in non-calcified plaque compared to placebo (−1.7 mm^3^ vs. 2.6 mm^3^; *P* = .044) 33% relative reduction in non-calcified plaque progression
PCSK9 inhibition^85^	Presence of vulnerable plaque	98 patients with 136 vulnerable plaques (lesions with HU < 50)	6 months	Increase in minimal Hounsfield Unit value (39.1 ± 8.1 HU to 84.9 ± 31.4 HU, *P* < .001) Reduction in remodelling index (1.29 ± 0.11 to 1.19 ± 0.10, *P* < .001)
Icosapent ethyl^86,87,89^	CAD and elevated triglyceride levels	31 patients receiving icosapent ethyl 37 receiving placebo	18 months	Reduction in low-attenuation (−17%; *P* = .0061), fibro-fatty (−34%; *P* = .0002), and fibrous plaque (−20%; *P* = .0028) Significant benefits in coronary physiology as assessed by change in fractional flow reserve No change in calcified plaque
Colchicine^88^	Recent ACS <1 month	40 patients receiving colchicine 40 controls	13 months	Reduction in low-attenuation plaque volume (15.9 mm^3^ vs. 6.6 mm^3^; *P* = .008) No reduction in total plaque progression
Diet intervention^91^	Non-obstructive CAD (<70%)	45 in diet intervention 44 controls	15 months	Reduction in non-calcified plaque compared to placebo (−51.3 mm^3^ vs. −21.3 mm^3^; *P* = .045) No reduction in total or calcified plaque progression

ACS, acute coronary syndrome; CAD, coronary artery disease; HIV, human immunodeficiency virus; PCSK9, proprotein convertase subtilisin-kexin type 9.

**Figure 3:**
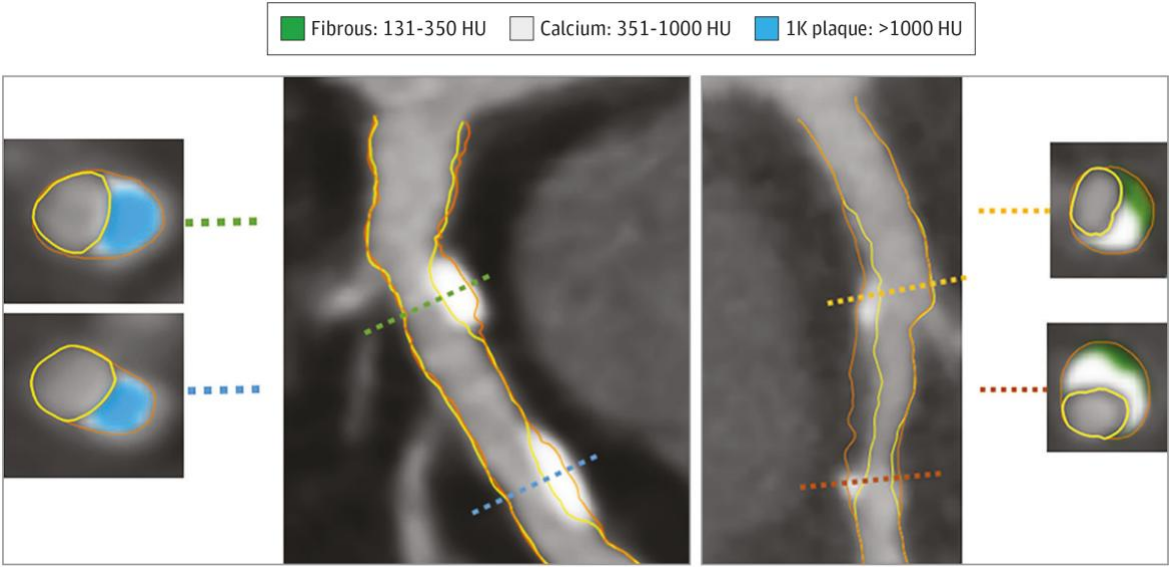
Example of 1K plaque. The artery segment in the left panel (*A*) shows two lesions composed of 1K plaque without non-calcified plaque. Cross-sectional examples are shown with 1K plaque. The artery segment in (*B*) shows calcifications between 351 and 1000 HU intermingled in non-calcified plaque. Two cross-sections show 351 to 1000 HU calcium together with fibrous plaque tissue. HU, Hounsfield units. Adapted from van Rosendael *et al.*^92^

The hypothesis that plaque transformation from non-calcified to calcified is a risk-lowering phenomenon is supported by three observations. First, high-density calcium is associated with lower risk for future coronary syndrome as compared with non-calcified plaque (*Figure 3*).^92^ Second, statins (as well as other medications and lifestyle interventions) provoke a more rapid transformation of low-density non-calcified plaque to high-density calcium. Third, the higher the density of calcified plaque at baseline, the lower the plaque progression rates of these lesions.^90^ The notion that statins increase coronary calcium explains why serial CACS is not useful in patients receiving preventive medical therapy, and suggests that serial CCTA-guided atherosclerosis evaluation can enhance our understanding of the dynamic nature of CAD that predisposes an individual to plaque vulnerability.

### Treating atherosclerosis, not stenosis or ischaemia, improves patient outcomes

To date, only one large-scale randomized controlled trial has demonstrated the use of non-invasive CAD imaging to guide therapy in a manner that reduces MACE. The SCOT-HEART trial randomized patients with stable chest pain to standard care vs. CCTA-guided care, which resulted in a 40% reduction in death from CHD or myocardial infarction at 5-year follow-up.^93^ Importantly, nearly 50% of events occurred in patients with non-obstructive stenosis which would not have been detected by ischaemia imaging modalities. The direct visualization of atherosclerosis by CCTA compared with standard care led to higher rates of statin and aspirin prescription, and more appropriate prescriptions targeting patient with actual disease, which can explain the benefit observed in SCOT-HEART.^94,95^ The results from SCOT-HEART^96^ are consistent with findings from prior studies—including COURAGE,^97^ BARI 2D,^98^ ORBITA,^99^ and ISCHEMIA^100^—wherein a stenosis- or ischaemia-guided approach did not improve prognosis, highlighting the inadequacy of emphasizing these downstream sequelae of atherosclerosis over the disease itself.

### Role of coronary computed tomography angiography as a ‘gatekeeper’ to downstream unnecessary procedures

**Figure 4:**
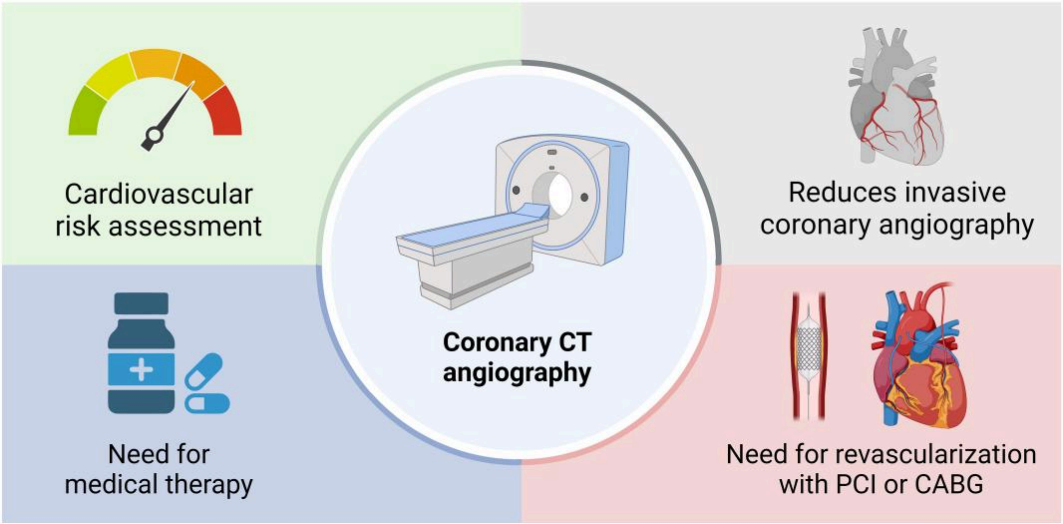
Multidimensional role of coronary computed tomography (CT) in coronary artery disease. Coronary CT angiography can refine risk stratification, determine need for medical therapy, can reduce unnecessary invasive coronary angiography and determined the need for revascularization with percutaneous coronary intervention (PCI) or coronary artery bypass grafting (CABG)

An important aim of non-invasive diagnostic testing suspected CAD is to identify those with ischaemia who might benefit from coronary revascularization (*Figure 4*). While ischaemia testing has held a historically prominent position in the diagnostic work-up of symptomatic suspected CAD, its benefit has been proven for symptom relief but not risk reduction. Despite its widespread use, ischaemia testing has limited diagnostic performance. Amongst 398 978 patients undergoing elective invasive angiography, only 37.6% of patients had obstructive CAD.^101^ Notably, 83.9% of patients underwent previous non-invasive imaging, mainly functional imaging by stress testing.^102,103^ This ischaemia-driven referral was driven, in part, by results of the FAME trials showing ischaemia-guided coronary intervention to confer symptomatic benefit.^104,105^ However, in the study by Patel *et al.*,^101^ the yield of obstructive CAD on invasive coronary angiography (ICA) was low, with nearly two of three patients referred for ICA identified as having no actionable CAD. This raises significant concerns about the ability of current testing methods to correctly identify patients referred for ICA.

A growing body of evidence indicates that, at present, patients referred for ICA are lower risk and possess lower prevalence of ischaemia and ischaemic burden, which is also observed in numerous studies showing the proportion of patients with ischaemia has decreased considerably over the past years.^106,107^ This trend towards lower disease prevalence results in a reduction in specificity and positive predictive value creating more false-positive findings. The high negative predictive value of CCTA renders it an ideal tool for excluding obstructive CAD (i.e. ≥50% or ≥70% diameter stenosis) with near to absolute certainty in low-to-intermediate risk populations.^108^ As such, CCTA can serve as an effective gatekeeper for avoidance of ICA in low-to-intermediate populations with mild or no CAD. In this regard, the CONSERVE trial investigated normalcy rates, number of invasive procedures and outcomes in patients in whom decision to proceed to ICA was informed by CCTA compared to a direct referral strategy.^42^ The authors observed that ICA was effectively avoided in 77% of patients by CCTA, with a concomitant cost reduction of 57%. ICA avoidance was safe, with MACE (4.6% in both arms) similar between groups, whereas the ICA normalcy rates were significantly lower in the CCTA arm (25% vs. 61%, *P* < .001).^42^ Similar findings were seen in the DISCHARGE trial, where no difference in MACE was observed between a CCTA and ICA strategy (2.1% vs. 3.0%; *P* = .10).^109^ Importantly, frequency of procedure-related complications was a three-fold lower in patients undergoing CCTA vs. ICA (0.5% vs. 1.9%; odds ratio 0.26; 95% CI 0.13–0.55).^96,110^

## The potential role of coronary computed tomography angiography in asymptomatic patients

### Relationship between cardiovascular risk factors and coronary computed tomography angiography-defined atherosclerosis in asymptomatic individuals

Elevated serum lipid levels (low-density lipoprotein cholesterol, lipoprotein(a), triglycerides), inflammatory proteins (high-sensitivity C-reactive protein), glucose/glycated haemoglobin, and blood pressure are traditional CAD risk factors that increase the likelihood of atherosclerosis in the coronary arteries, and hence are important targets for ASCVD risk-lowering therapies. On a population level, significant associations have been observed between risk factors, atherosclerosis, and MACE.^111–113^ Coronary atherosclerosis is more prevalent in the presence of risk factors, but the association between risk factors and atherosclerosis demonstrates imprecision and high variability at the individual level. *Table 4* shows the risk ratios for the presence of any coronary atherosclerosis by CCTA and obstructive stenosis in a cohort of 2359 asymptomatic individuals from the Miami Heart study,^114^ ranging from one- to four-fold increases for obesity, hypercholesterolaemia, diabetes, current smoking, family history with premature CAD, and hypertension. On an individual patient level, clinical risk estimates have proven to be a poor predictor of the actual burden of coronary atherosclerosis. As an example, among 10 000 patients without prior CAD from the CONFIRM registry, although not all asymptomatic, only slightly more atherosclerosis was present in patients with diabetes compared to those without diabetes, when matched for age, sex, and the other classic cardiovascular risk factors: normal, non-obstructive, and obstructive CCTA was observed in 28% vs. 36% (*P* < .001), 35% vs. 37% (*P* = .04), and 37% vs. 27% (*P* < .001), respectively.^57^ Moreover, CCTA has revealed that the notion that diabetes is equivalent to established atherosclerosis is inaccurate, given the ∼30% of patients with diabetes who have no identifiable atherosclerosis.^115–117^ An overview of atherosclerosis severity per specific risk group is provided in *Table 5*. Overall, a normal CCTA was observed in 28% to 63% of patients, while obstructive CAD was present in 5% to 26% of patients, depending on the cohort.

**Table 4:** Associations between risk factors and coronary computed tomography angiography-defined atherosclerosis in asymptomatic individuals (Miami Heart Study)

	Presence of atherosclerotic plaque (univariate OR with 95% CI)	≥50 stenosis (univariate OR with 95% CI)
Obesity	2.59 (2.08–3.24)	3.79 (2.10–6.82)
Hypercholesterolaemia	2.48 (2.09–2.95)	2.40 (1.59–3.62)
Diabetes	2.28 (1.67–3.11)	3.92 (2.57–5.97)
Current smoking	2.38 (1.43–3.96)	2.72 (1.36–5.43)
Family history with premature CAD	1.10 (0.80–1.52)	1.08 (0.55–2.09)
Hypertension	2.24 (1.90–2.64)	2.90 (1.93–4.36)

Data from the Miami Heart Study.^114^

CAD, coronary artery disease; CCTA, coronary CT angiography; OR, odds ratio; CI, confidence interval.

**Table 5:** Coronary atherosclerosis presence by risk factor in individuals without prior coronary artery disease

Risk factor	*n*	Symptoms	Age (years)	Normal CCTA (%)	Non-obstructive CAD (%)	Obstructive CAD (%)
Familial hypercholesterolaemia^118^	50	Asymptomatic	48	50	22	26
Hypertension^119^	1434	38% asymptomatic	57	44	38	18
Diabetes^57^	3370	30% asymptomatic	61	28	35	37
Metabolic syndrome^120^	690	39% asymptomatic	58	53	32	15
Asymptomatic US population^114^	2359	Asymptomatic	53	51	43	7
Asymptomatic US population, ASCVD score 7.5%–20%^114^	219	Asymptomatic	–	25	61	14
Asymptomatic South Korean population, Lp(a) quartile 1^121^	1804	Asymptomatic	53	66	29	5
Asymptomatic South Korean population, Lp(a) quartile 4^121^	1798	Asymptomatic	55	63	30	7

ASCVD, atherosclerotic cardiovascular disease; CAD, coronary artery disease; CCTA, coronary CT angiography; Lp(a), lipoprotein(a).

### Coronary computed tomography angiography-defined atherosclerosis for prediction of major adverse cardiovascular events in asymptomatic individuals

Since CCTA is not routinely recommended in cardiovascular risk screening in asymptomatic patients,^3,4^ there are relatively few large studies evaluating the value of CCTA for cardiovascular risk stratification. In two studies from CONFIRM with 2 and 6 years of follow-up, restricted to asymptomatic patients without known CAD, CCTA did not add any relevant incremental prognostic value beyond CACS when added to a model with clinical risk factors.^56,122^ In a more recent study from the Copenhagen General Population Study, the prognostic value of CCTA was investigated in 9533 asymptomatic patients with a mean age of 60 years.^123^ In this population study, subclinical atherosclerosis was found in 61% of men and 36% of women. After adjustment for sex, age, and cardiovascular risk factors, the presence of subclinical obstructive atherosclerosis was associated with a more than 8-fold up to 12-fold increased risk of myocardial infarction, depending on the extent of the CAD. Furthermore, presence of a high-risk plaque feature (spotty calcification, napkin-ring sign, or non-calcified plaque) was associated with a three-fold increased risk of myocardial infarction. Although CACS >300 was also associated with a seven-fold risk increase of myocardial infarction, the study did not investigate the incremental prognostic value of CCTA beyond clinical risk factors or CACS.

While CACS has shown incremental value and might be considered in asymptomatic individuals around risk thresholds, it remains unknown whether CCTA can further improve risk stratification in asymptomatic patients, considering the limited amount of data available.^3,4^ The fact that relative reductions in events in the SCOT-HEART trial were similar in those with non-cardiac chest pain, suggests a directionally similar effect, but further studies are highly needed.^93^

### Coronary computed tomography angiography-based atherosclerosis assessment in apparently healthy individuals to guide preventive therapy

To date, results of randomized studies evaluating the use of CT in asymptomatic, apparently healthy individuals have been less robust. The St. Francis Heart Study randomized 1005 asymptomatic subjects with CACS >80th percentile for age and sex to atorvastatin vs. placebo.^124^ At 4.3 years, no differences were seen in patients for CACS progression, with treatment failing to reduce MACE. Notably, patients with CACS >400 did experience a reduction in events (8.7% vs. 15.0%, *P* = .046).^124^ While this was not prespecified, it nevertheless raises the possibility of benefit of atherosclerosis screening by CT if proper identification of high-risk individuals can be achieved.

In 2022, the results of DANCAVAS were reported for men 65–74 years of age who underwent non-contrast CT for CACS, ankle-brachial indices and cholesterol and diabetes serum biomarker diagnosis.^125^ In this population-based screening study, a total of 46 611 men underwent randomization. For a primary endpoint of all-cause mortality at 5 years, the study was not statistically significant but directionally suggested possible benefit of screening with CT (HR 0.95, 95% CI 0.90–1.00, *P* = .06). In a prespecified subgroup analysis, potential benefits were seen in those of younger age (65–69 years), with an 11% lower rate of death (*P* = .007) and a 7% lower rate of a primary composite endpoint of death, stroke, or myocardial infarction (*P* = .016).

That both the St. Francis Heart Study and the DANCAVAS trials used CACS over CCTA precludes assessment of the value of non-calcified plaque for better identification and image-guided treatment. To date, only one study has been reported to evaluate the use of CCTA of screening—the FACTOR-64 study.^126^ In this study of 900 patients randomized to CCTA or no CCTA, patients with diabetes were recommended for treatment of cholesterol and diabetes and lifestyle based upon CCTA findings of stenosis and without atherosclerosis evaluation. The endpoint—all-cause mortality, non-fatal myocardial infarction, or hospitalization for unstable angina—did not differ between the CCTA and no CCTA arms. Whether latest generation quantitative atherosclerosis evaluation tools may have influenced these findings requires future study.

## Future directions

### Atherosclerosis evaluation by coronary computed tomography angiography encourages a shift in the preventive care paradigm from population-based to personalized

CCTA has the potential to individualize risk assessment by allowing for direct visualization of atherosclerosis in a non-invasive manner. It offers the ability to quantify plaque burden and assess plaque morphology, as well as the ability to define high-risk measures of plaque and vascular morphology. Diameter stenosis, plaque burden, and specific plaque phenotypes, such as non-calcified and, in particular, low-attenuation plaque, are considered some of the most potent markers for future MACE and carry the potential to be the standard of care for risk assessment. Temporal changes in plaque burden and morphology further refine risk stratification, offering the impetus to achieve personalized medicine.

**Figure 5:**
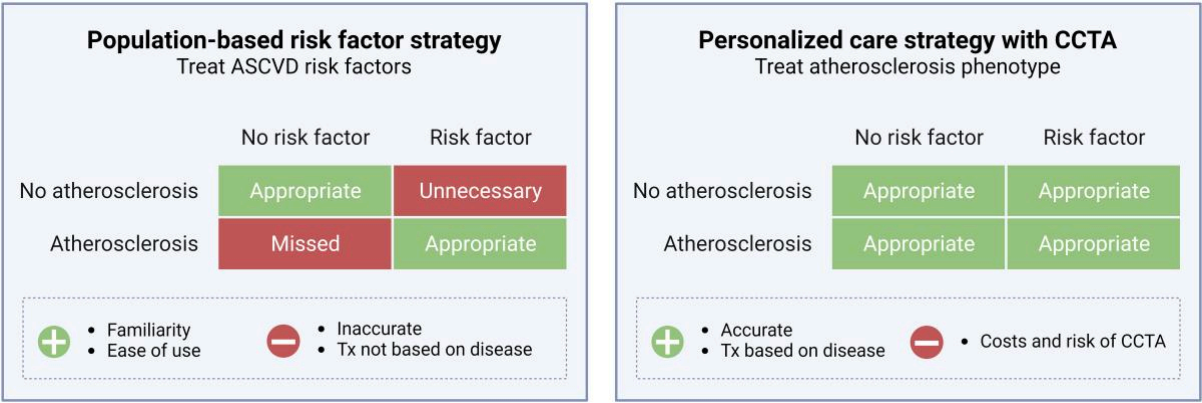
Comparison of population risk-based prevention with personalized prevention strategies using coronary computed tomography angiography. Differences in a population-based approach based on risk factors (e.g. commonly used ASCVD risk scores) and a personalized care strategy based on the actual disease phenotype observed with CCTA. In a population-based risk factor strategy, patients are treated based on the presence of risk factors. If the presence of risk factors aligns with the presence of atherosclerosis, patients are treated appropriately. In a population-based risk factor strategy, patients without risk factors but with presence of atherosclerosis are missed, while patients with risk factors but without atherosclerosis (above a certain age threshold) are unnecessarily treated. In a personalized care strategy with CCTA, patients can receive appropriate therapy based on their individual atherosclerosis phenotype. ASCVD, atherosclerotic cardiovascular disease; CCTA, coronary computed tomography angiography; Tx, treatment

Nevertheless, current risk prediction tools are traditionally based upon traditional cardiac risk factors such as hypertension, diabetes, hypercholesterolaemia, age, sex, and a few tools also incorporate C-reactive protein and lipoprotein(a).^1–5^ These risk factors of atherosclerosis reflect population-level factors associated with CAD that correlate imprecisely to any given individual’s disease. Although these risk tools are accessible, they fail to accurately predict future adverse events in asymptomatic individuals with diabetes and symptomatic patients, cannot discriminate individuals with vs. without high-risk CAD, and may give false security to those with no risk factors but significant CAD (*Figure 5*).

Numerous studies have shown a CACS =0 to be a potent negative risk marker in general populations, one that downgrades individual risk and identifies individuals with a negligible risk of future MACE outcome.^127–133^ Yet, contemporary research has demonstrated the inadequacy of CAC scoring for identifying the totality of at-risk individuals. Coronary artery calcium score data from population-based studies have revealed up to 1 in 4 younger individuals who will suffer MACE will have a CACS =0 at the time of imaging,^16,134^ emphasizing the urgency of improved approaches to pinpoint at-risk individuals. That CACS has been prognostically useful in large-scale outcomes studies is unsurprising, as many who have CAC also possess higher-risk non-calcified plaques. However, as adverse atherosclerotic plaque morphology is defined by lower-density non-calcified plaques, CCTA may represent an improved approach to evaluate individuals in a personalized fashion for total plaque burden, as well as makeup of plaque morphology, which has been shown to be a better guide for risk stratification.^62^

Visualization of atherosclerosis as the primary heart disease process holds the potential to shift our current preventive care paradigm emphasis on population-based risk factors to individualized disease burden and type in a manner that may guide therapeutic decision making of medical therapy and lifestyle interventions (*Figure 4*). Coronary atherosclerosis is a single trackable metric that represents an individual’s lifelong exposure to all known and unknown risk factors. In this manner, clinical evaluation of atherosclerosis may both allow for deferral of initiating lifelong therapy or escalation of medical therapy based upon disease or changes in disease, thereby increasing the prognostic benefit of our therapeutics.

### Perspectives for future clinical trials

Over the last 20 years, CCTA has witnessed important advances in its technology, with improved spatial and temporal resolution, larger volume coverage, and lower radiation exposure and contrast requirements. In conjunction with continued automation in quantitative CCTA analysis with AI-supported algorithms,^62–64,135–141^ allowing for reliable and reproducible identification of coronary plaque volume and potential high-risk plaque, atherosclerosis assessment by CCTA has the potential to further improve cardiovascular risk stratification. However, large-scale prospective studies comparing atherosclerosis assessment by CCTA to the clinical standard of care are highly needed before widespread implementation in clinical practice. Furthermore, the direct visualization of coronary atherosclerosis by CCTA allows for the evaluation CAD burden and morphology and, given its non-invasive nature, allows for serial assessment to identify temporal changes in an individual’s disease process.^79^ Quantitative CCTA evaluation of atherosclerosis^135–141^—coupled with the near universal availability of CT scanners—may provide a useful tool to evaluate the effectiveness of medical therapy and lifestyle interventions over time integrating large-scale randomized controlled trial data with an ‘*n* of 1’ approach that is dictated by actual CAD compared to optimization of imprecisely associated risk factors within a single individual (*Table 5*). In the meantime, assessment of both stenosis and a visual assessment of plaque burden such as the segment involvement score, as advocated by CAD-RADS 2.0,^142^ could significantly improve ASCVD risk stratification in current clinical practice.

To date, most CCTA studies have been performed in symptomatic patients with stable chest pain. Given its safety and ease of performance, use of CCTA has been advocated by some to serve as a ‘mammogram of the heart,’ i.e. to leverage its use in screening large populations for early identification, risk stratification, and treatment. While no study to date has been performed to directly addressing the screening indication of CCTA, in aggregate, the previously performed large-scale randomized trials offer hypothesis-generating results that image-guided screening by CCTA may serve as an effective tool to identify asymptomatic individuals at risk of MACE and to guide judicious use of preventive therapies. These studies also highlight limitations that must be considered for expansion of CCTA use in screening populations:

- *Tiered treatment commensurate to individualized disease burden*. In the prior studies, there has been ambiguity in treatment recommendations based upon CT for atherosclerosis or stenosis. Notably, these trials were performed at a time when effective medical prevention of CAD primarily consisted of statins, with no access to contemporary agents such as PCSK9i monoclonals, inclisiran, icosapent ethyl, bempedoic acid, low-dose rivaroxaban, GLP1 receptor agonists, GLP1/GIP agonists, SGLT2 inhibitors, SGLT1/2 inhibitors, and upcoming therapies targeting inflammation and lipoprotein(a). A contemporary assessment of CT-guided vs. non-CT-guided treatment leveraging the entirety of contemporary medical therapy is needed.
- *Appropriate clinical endpoints.* Prior studies have either evaluated endpoints of all-cause mortality (with its intrinsic competing risks from non-cardiovascular deaths that cannot be influenced by treating CAD) or ‘soft’ endpoints comprising revascularization.
- *Appropriate CAD measurements.* To date, only FACTOR-64 has evaluated the effectiveness of CCTA for screening but was performed at a time when CCTA was primarily used for stenosis severity evaluation. No study to date has leveraged total atherosclerotic plaque burden and type to guide therapy.
- *Adequate sample size and follow-up.* Previous studies have been underpowered to observe realistic clinical differences between image-based and non-image-based arms. To address this, future trials would be ideally event-driven along with minimal treatment durations. These primary endpoints should also emphasize important patient-centric clinical outcomes that are of uniform importance to patients and will require very large sample sizes and adequate follow-up time to achieve. The SCOT-HEART2 trial (ClinicalTrials.gov: NCT03920176), the DANE-HEART trial (ClinicalTrials.gov: NCT05677386), and the TRANSFORM trial (ClinicalTrials.gov: NCT06112418) will each have an estimated sample size of 6000 to 7500 patients with a follow-up duration of 4–5 years, which optimizes the chance of demonstrating clinically significant benefit.

## Conclusions

Over two decades, CCTA has advanced our understanding of coronary biology in a manner that can now effectively pinpoint risk and guide therapy in a personalized fashion (*Graphical Abstract*). Adoption of this precision heart care approach may be beneficial, but large-scale trials will be required to demonstrate the benefit with clinical outcomes. Given that the most successful preventive care paradigms have relied upon advanced imaging for direct visualization—including mammograms, colonoscopies, and lung CT for early identification and treatment of breast, colon, and lung cancer, respectively—it is conceivable that emulation of these approaches for CAD prevention over sole use of indirect risk factors may hold the potential to improve patient outcomes by combining large-scale RCT evidence with clinic-based ‘*n* of 1’ approaches to personalized care.

## Declarations

### Data Availability

No data were generated or analysed for or in support of this article.
