# Correction to: Atherosclerosis evaluation and cardiovascular risk estimation using coronary computed tomography angiography

**DOI:** 10.1093/eurheartj/ehae318

**Published:** 2024-05-15

This is a correction to: Nick S Nurmohamed et al., Atherosclerosis evaluation and cardiovascular risk estimation using coronary computed tomography angiography, *European Heart Journal*, 2024; ehae190, https://doi.org/10.1093/eurheartj/ehae190.

In the section ‘Serial coronary computed tomography angiography studies investigating the impact of medical therapy’ the text “The hypothesis that plaque transformation from calcified to non-calcified is a risk-lowering phenomenon is supported by three observations” has been corrected to read “The hypothesis that plaque transformation from non-calcified to calcified is a risk-lowering phenomenon is supported by three observations”.

